# Blood myeloid cells differentiate to lung resident cells and respond to pathogen stimuli in a 3D human tissue-engineered lung model

**DOI:** 10.3389/fbioe.2023.1212230

**Published:** 2023-07-07

**Authors:** Mandi M. Roe, Taylor Do, Sean Turner, Allison M. Jevitt, Magdalena Chlebicz, Karley White, Antonius G. P. Oomens, Susannah Rankin, Susan Kovats, Heather Gappa-Fahlenkamp

**Affiliations:** ^1^ Kovats Lab, Oklahoma Medical Research Foundation, Arthritis and Clinical Immunology Program, Oklahoma City, OK, United States; ^2^ Fahlenkamp Lab, School of Chemical Engineering, Oklahoma State University, Stillwater, OK, United States; ^3^ Rankin Lab, Oklahoma Medical Research Foundation, Cell Cycle and Cancer Biology Program, Oklahoma City, OK, United States; ^4^ Oomens Lab, Department of Veterinary Pathobiology, College of Veterinary Medicine, Oklahoma State University, Stillwater, OK, United States; ^5^ Department of Microbiology and Immunology, University of Oklahoma Health Sciences Center, Oklahoma City, OK, United States

**Keywords:** lung model, monocyte, macrophage, differentiation, innate immune, respiratory syncytial virus

## Abstract

**Introduction:** Respiratory infections remain a leading global health concern. Models that recapitulate the cellular complexity of the lower airway of humans will provide important information about how the immune response reflects the interactions between diverse cell types during infection. We developed a 3D human tissue-engineered lung model (3D-HTLM) composed of primary human pulmonary epithelial and endothelial cells with added blood myeloid cells that allows assessment of the innate immune response to respiratory infection.

**Methods:** The 3D-HTLM consists of small airway epithelial cells grown at air-liquid interface layered on fibroblasts within a collagen matrix atop a permeable membrane with pulmonary microvascular endothelial cells layered underneath. After the epithelial and endothelial layers had reached confluency, an enriched blood monocyte population, containing mostly CD14^+^ monocytes (Mo) with minor subsets of CD1c^+^ classical dendritic cells (cDC2s), monocyte-derived dendritic cells (Mo-DCs), and CD16^+^ non-classical monocytes, was added to the endothelial side of the model.

**Results:** Immunofluorescence imaging showed the myeloid cells migrate through and reside within each layer of the model. The myeloid cell subsets adapted to the lung environment in the 3D-HTLM, with increased proportions of the recovered cells expressing lung tissue resident markers CD206, CD169, and CD163 compared with blood myeloid cells, including a population with features of alveolar macrophages. Myeloid subsets recovered from the 3D-HTLM displayed increased expression of HLA-DR and the co-stimulatory markers CD86, CD40, and PDL1. Upon stimulation of the 3D-HTLM with the toll-like receptor 4 (TLR4) agonist bacterial lipopolysaccharide (LPS), the CD31^+^ endothelial cells increased expression of ICAM-1 and the production of IL-10 and TNFα was dependent on the presence of myeloid cells. Challenge with respiratory syncytial virus (RSV) led to increased expression of macrophage activation and antiviral pathway genes by cells in the 3D-HTLM.

**Discussion:** The 3D-HTLM provides a lower airway environment that promotes differentiation of blood myeloid cells into lung tissue resident cells and enables the study of respiratory infection in a physiological cellular context.

## 1 Introduction

Lower respiratory infections have been among the top five leading causes of death worldwide since 2000 (WHO), and the COVID-19 pandemic has further highlighted the need to understand how respiratory viruses elicit and subvert responses generated by multiple epithelial cell types and resident immune cells of the lung. Tissue-engineered lung models focus on modeling the human lower respiratory system to better understand the pulmonary immune response to respiratory pathogens. These models range in complexity based on cell types used and dimensionality, but often use immortalized cell lines, which are limited in how well they mimic a healthy lung environment (Joris et al., 2013; He et al., 2021). Lung organoids are another commonly used primary cell lung model that demonstrate epithelial cell composition and structure similar to tissues *in vivo,* yet lack appropriate polarity such as an air-liquid interface (Sachs et al., 2019; Salahudeen et al., 2020). Our previous tissue-engineered model incorporated this polarity with small airway epithelial cells at air-liquid interface layered on a collagen matrix (Bhowmick et al., 2018). To model *in vivo* interactions of multiple human cell types in the lower respiratory tract, we have now developed a three-dimensional human tissue-engineered lung model (3D-HTLM) composed of primary epithelial and endothelial cells surrounding a collagen scaffold seeded with fibroblasts. To this structure, we added myeloid cells, which constitute significant populations of lung resident immune cells in homeostasis. This second-generation 3D-HTLM consists of primary small airway epithelial cells (SAECs) grown at an air-liquid interface (ALI), pulmonary microvascular endothelial cells (HPMEC), pulmonary fibroblasts (HPF) within a collagen matrix, and enriched blood monocytes (Mo) including minor populations of dendritic cells (DCs) and monocyte-derived DCs (Mo-DCs).

The diverse cells in the lower airway environment work together to promote immunity. As the interactive barrier between peripheral blood and lung tissue, HPMECs play a role in the recruitment of myeloid cells (Teijaro et al., 2011; Viemann et al., 2011). SAECs promote barrier function of the small airways by forming tight and adherens junctions, which establish polarity and separate the apical and basolateral surfaces of the lung (Strengert and Knaus, 2011). SAECs grown at ALI with a collagen scaffold have increased expression of differentiation markers found in the human lung compared with SAECs grown at ALI without a collagen scaffold (Bhowmick et al., 2018). Furthermore, GM-CSF released by alveolar epithelial cells is crucial to the immune response within the small airway as it instructs the development and maintenance of alveolar macrophages (AM) (Schneider et al., 2014; Gschwend et al., 2021). AM are the most abundant resident myeloid cell within the resting airway and function to sample inhaled antigens and maintain regulation and clearance of surfactant lipoproteins.

An important goal of this study was to determine if blood Mo and DCs added to the 3D-HTLM differentiate to acquire the phenotype and functional capacity of human lung tissue resident myeloid cells, including alveolar macrophages. AM develop from fetal precursors and are mostly self-renewing but can be diminished through age and infection (Lavin et al., 2014; Kopf et al., 2015; Lavin et al., 2015). Depleted AM populations can be replenished through an influx of blood monocytes that differentiate into cells phenotypically and transcriptionally resembling AM (van de Laar et al., 2016). Conventional DCs (cDCs) are present as minor populations of myeloid cells within the lung, but play an important role in pathogen sensing, cytokine production, and activation of the adaptive immune response to infection (Ainsua-Enrich et al., 2019). Other resident myeloid populations include monocytes and interstitial macrophages, which also promote pathogen responses (Chakarov et al., 2019; Aegerter et al., 2020; Mulder et al., 2021). Lung resident myeloid cells express multiple surface markers that are not seen in circulating myeloid cells, such as CD206, CD169, and CD163 (Yu et al., 2016; Patel et al., 2017; Patel and Metcalf, 2018; Evren et al., 2021). Monocytes and macrophages secrete pro- and anti-inflammatory cytokines upon bacterial or viral challenge (Rossol et al., 2011; Hijano et al., 2019).

We here demonstrate the generation of functional primary human epithelial and endothelial cell layers on a fibroblast-enriched collagen gel matrix to form a biomimetic human tissue lung model. SAECs grown at air-liquid interface formed a confluent layer, and the fully built 3D-HTLM showed increased trans-epithelial electrical resistance (TEER), indicating robust integrity. CD31^+^ HPMECs formed a confluent layer and upregulated the inflammatory marker ICAM-1 upon stimulation with the TLR4 agonist LPS. Blood myeloid cells added to the HPMEC side of the 3D-HTLM migrated throughout the model and acquired characteristics of lung tissue resident cells based on morphology and surface markers HLA-DR, CD169, CD206, and CD163, including a minor subset of cells characterized as AM (CD1c^−^CD14^lo^HLA-DR^+^CD169^+^CD206^+^CD71^+^). The myeloid cells within the 3D-HTLM responded to LPS stimulation with increased secretion of IL-10, IL-6, and TNFα, while costimulatory and phenotypic markers reflected levels inherent to each subset. Upon challenge with RSV, cells in the 3D-HTLM increased expression of genes involved with macrophage activation, viral sensing and antiviral activity, and IFN signaling. Herein, we demonstrate an *in vitro* lung model comprised of multiple primary cell types in a 3D structure that can be used to study the human lower airway response to respiratory pathogens.

## 2 Materials and methods

### 2.1 Collagen hydrogel preparation and fibroblast seeding

The primary cells used to build the models were kept as low passage number frozen stocks in liquid nitrogen. The 3D models were grown on Celltreat (Celltreat Scientific Products, Pepperell, MA) 24-well hanging cell culture inserts with a permeable 8 μM PET membrane. The collagen hydrogel was developed using previously defined methods (Gappa-Fahlenkamp and Shukla, 2009) (Derakhshan et al., 2018), and consisted of 64.5 vol% 3.1 mg/mL type 1 bovine collagen (Advanced BioMatrix; Carlsbad, CA), 8.1 vol% 10x M199 (Gibco), 13.3 vol% 0.1 N NaOH, and 14 vol% phosphate-buffered saline (PBS). Primary HPFs (PromoCell, Catalog #C-12360 Heidelburg, Germany) were thawed and then directly seeded within the collagen gel solution at a concentration of 75,000 cells/mL, and 0.150 mL of collagen solution was aliquoted to the inner well of the hanging cell culture inserts. Following 45 min of incubation at 37°C 5% CO_2_ concentration, complete SAEC medium (PromoCell, Catalog #C-21070 Heidelburg, Germany) was added to the bottom well chamber (1 mL), and 0.100 mL of medium was added to the top of the collagen gel. The hydrogels were incubated for 24 h before adding SAECs.

### 2.2 Culturing and addition of SAECs and HPMECs to 3D-HTLM

SAECs from a 67-year-old male, (PromoCell; Heidelburg, Germany) were thawed and expanded in number in 75 cm^2^ cell culture flasks in complete SAEC medium. Flasks were coated with a solution containing 25 μg/mL fibronectin in PBS with 2.5% of 3.1 mg/mL type 1 bovine collagen. Medium changes were performed every 72 h, and cells were grown until 80% confluent. 2 h prior to the addition of the SAECs to the models, the surface of the collagen hydrogels was coated with the fibronectin/collagen coating solution. Once confluent, the SAECs were lifted from the flask using Accutase-Solution (PromoCell) according to manufacturer’s instructions and seeded onto the surface of the collagen hydrogels in complete SAEC medium with a seeding density of 150,000 cells/cm^2^. SAECs were grown submerged in medium for 7 days.

**FIGURE 1:**
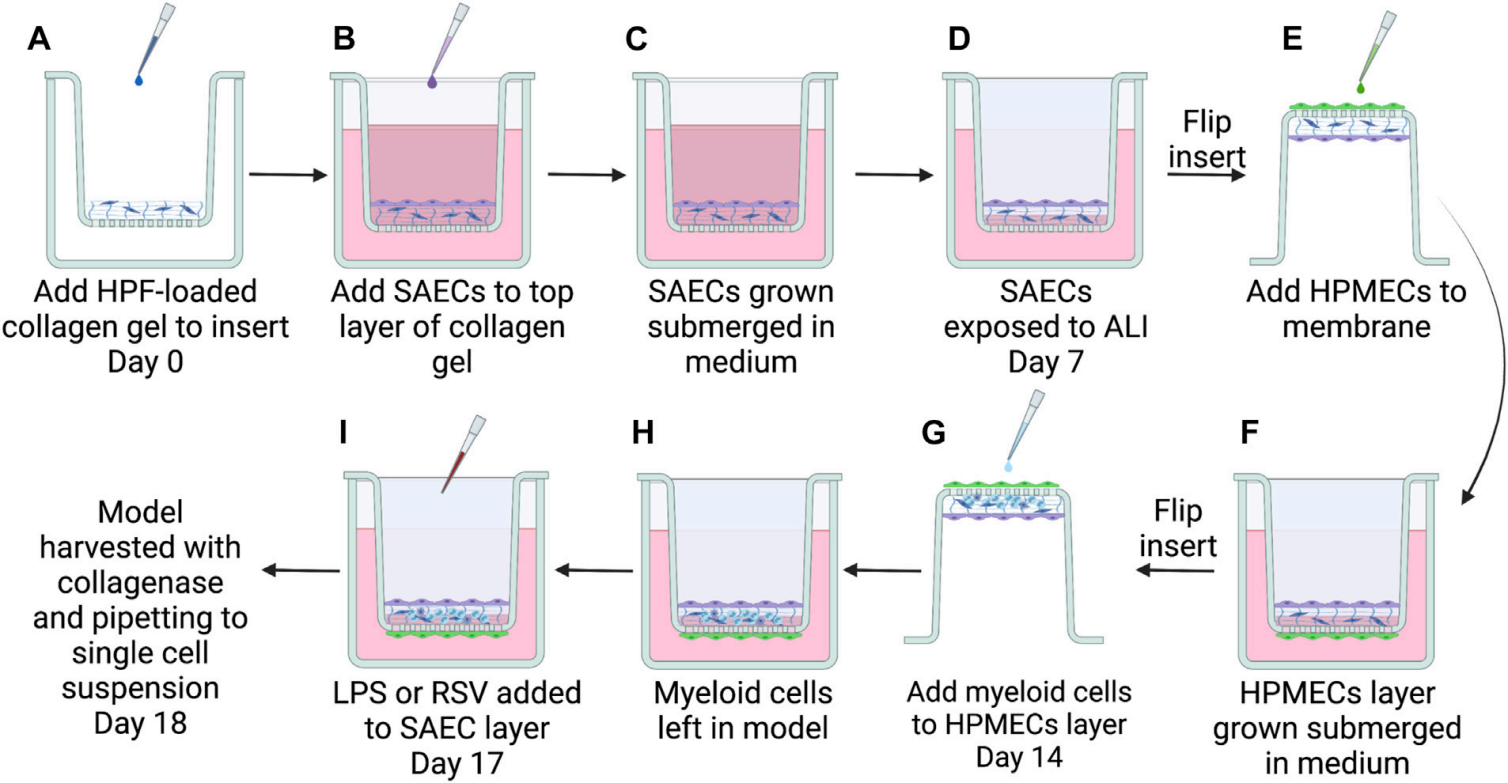
Steps to build the complete 3D-HTLM. **(A)** A collagen gel matrix loaded with HPFs was added to a hanging cell culture insert with a permeable 8 μM PET membrane. SAECs were then seeded on top of collagen gel **(B)** and grown submerged in cell culture medium for 7 days **(C)**. At day 7, SAECs were exposed to air-liquid interface (ALI) on day 7 **(D)** and HPMECs were seeded to underside of fibronectin-coated PET membrane **(E)**, given 4 h for attachment to membrane, then submerged in cell culture medium **(F)**. Seven days after seeding endothelial cells, enriched monocytes/DCs isolated from a human blood buffy coat were added to the endothelial layer of model **(G)**. Myeloid cells were allowed 4 h for migration into cellular matrix before the insert was resubmerged in medium with epithelial cells at ALI **(H)** and incubated 4 days for differentiation within the model. **(I)** 24 h before harvest, models were stimulated with 1 μg/mL LPS or infected with RSV MOI = 1. Graphic made with BioRender.

One week after seeding SAECs, the models were brought to air-liquid interface (ALI) by removing the medium from the top of the gels. The cell culture inserts were then inverted (Figure 1) into 12-well cell culture plates and coated with 0.075 mL of 25 μg/mL fibronectin for 1 h prior to adding HPMECs. Before addition to the model, HPMECs from a 70-year-old female (PromoCell) were thawed and expanded in number in complete Endothelial Cell MV2 medium (PromoCell, Catalog #C-22022) in 75 cm^2^ cell culture flasks, coated with 25 μg/mL fibronectin, and grown until 80% confluent. HPMECs were lifted from the flasks using Accutase-Solution and seeded onto the bottom side of the cell culture insert membrane with a seeding density of 150,000 cells/cm^2^. Models were incubated for 4 h before returning cell culture inserts to the 24-well plate, submerging the endothelial layer in medium. During the 4 h incubation period, the medium was changed to basal SAEC medium completed with Endothelial Cell MV2 Supplement Mix (PromoCell) (referred to as ALI media) and added to a level that maintained ALI for the epithelial layer (0.400 mL). Models were incubated for 1 week at ALI to form confluent endothelial and epithelial layers (Figure 1).

### 2.3 TEER

Trans-epithelial electrical resistance (TEER) (Ω∙cm^2^) was measured using an EVOM3 (World Precision Instruments). To determine the contribution to barrier integrity of each layer of the 3D-HTLM, TEER was measured for collagen hydrogels, HPFs seeded collagen hydrogels, HPFs seeded collagen hydrogels with SAECs, and HPFs seeded collagen hydrogels with SAECs and HPMECs. Measurements were taken at each medium change (every 36 h) for 13 days. The values were normalized to the resistance of a blank cell culture insert with medium following the equation:

$$TEER=\left(R_{sample}-R_{control}\right)\times A$$

where R_sample_ is the measured resistance of the experimental sample (Ω), R_control_ is the arithmetic mean of the blank cell culture insert (Ω), and A is the area of the PET membrane in the cell culture insert (0.33 cm^2^).

### 2.4 Characterization of SAECs, HPMECs, and HPFs

Immunofluorescence (IF) staining of the model lacking myeloid cells was performed separately on the endothelial and epithelial layers. Complete models were fixed with 4% paraformaldehyde (PFA). Hydrogels containing the epithelial layer and fibroblasts were then removed from the PET membrane and probed for CK-14 (1:50) followed by a secondary anti-mouse Ig PE. Samples were counterstained using ProLong antifade mountant with NucBlue (ThermoFisher Catalog #P36983). Samples were imaged with a Zeiss LSM 980 with an Airyscan two confocal microscope.

For sectioning to view all layers, models were fixed overnight in 4% PFA, then washed with PBS. The PBS was removed from the top portion of the transwell and replaced with warm 4% agarose and allowed to solidify. Next the PBS was removed from the bottom of the transwell, and the PET membrane with the agarose plug was removed from the hanging insert. 4% agarose was added to the bottom of the membrane. After solidifying, agarose was trimmed down and embedded in paraffin. Following processing, sectioning, deparaffinizing, and antigen retrieval, transverse sections were probed with Abs for CD31 (1:50), CD45 (1:100), CD14 (1:100), and Sytox deep red nuclear dye (Invitrogen, cat# S11381) to visualize endothelial and myeloid cells (Supplementary Table S1). Stained sections were preserved with ProLong Diamond Antifade mountant (Invitrogen, Cat#P36965). Alternatively, some deparaffinized sections were used for H&E staining. H&E sections were imaged with a Zeiss Axiovert 200 m inverted microscope with ORCA Fusion CMOS camera or a Nikon Eclipse Ni-E epifluorescence microscope. IF of models with RFP-RSV was done with models fixed with 4% PFA and whole mounted on a slide. Models were stained with DAPI and preserved with ProLong Diamond Antifade mountant. Slides were imaged with a Zeiss Axiovert 200M.

### 2.5 Isolation of monocytes and dendritic cells

Leukocyte buffy coats from anonymous donors were purchased from the Oklahoma Blood Institute. The OMRF Institutional Review Board approved the study. Donors were male or female between 37–69 years old. PBMC were isolated using density gradient centrifugation in Ficoll-Paque PLUS (GE Healthcare). Once isolated, the cell solution was negatively selected to enrich CD14^++^ CD16^−^, CD14^+^ CD16^++^, and CD14^++^ CD16^+^ myeloid cells, a population that also included minor subsets of cDC1s and cDC2s, using a Pan Monocyte Isolation Kit (Miltenyi Biotec, Catalog #130-096-537) and magnetic separation columns.

### 2.6 Addition of myeloid cells to 3D-HTLM

Frozen vials of myeloid cells (monocytes and DCs) were thawed and directly added to the 3D-HTLM on the endothelial interface 7 days after the addition of HPMECs. Cell culture inserts were temporarily inverted into 12-well cell culture plates and monocytes were added at a density of approximately 758,000 cells/cm^2^, (250,000 cells/model well) to the endothelial layer of the tissue models and incubated for 4 h before being returned to the 24-well plate with the endothelial cells submerged and the epithelial cells at ALI (Figure 1). Complete models with myeloid cells were incubated 4 days in a cytokine-enriched medium containing GM-CSF (10 ng/mL), FLT3L (33 ng/mL), and SCF (20 ng/mL) in basal SAEC medium complete with Endothelial MV2 Supplement Mix. While it was not possible to quantify the number of myeloid cells that entered the model, an average of 140,000 myeloid cells/model well was recovered after model digestion on day four post myeloid cell addition.

### 2.7 Monocyte only culture

Blood myeloid cells (monocytes and DCs) were plated at 2 × 10^6^ cells per tissue culture well in a 24-well or 96-well cell culture plate in the basal SAEC medium completed with Endothelial Cell MV2 Supplement Mix with the addition of GM-CSF (10 ng/mL), FLT3L (33 ng/mL), and SCF (20 ng/mL) for 4 days to compare the differentiation of myeloid cells in standard tissue culture conditions in the absence of the other cells of the model. Alternatively, blood myeloid cells were cultured for 4 days in ALI media with 50% 3D-HTLM conditioned medium obtained from unstimulated models lacking myeloid cells, supplemented by GM-CSF, FLT3L, and SCF.

### 2.8 Activation of 3D-HTLM

To test immunocompetency of the 3D-HTLM, the TLR4 agonist bacterial lipopolysaccharide (LPS) (Sigma, cat #L4391) or RSV A2 was added to the fully developed model. Three days after addition of myeloid cells, LPS (30 ng in 30 μL) was added to the surface of the epithelial layer of each well of the model, and the model was incubated for 24 h before digestion. Alternatively, RSV strain A2 or a recombinant RFP-RSV, constructed by Dr. Oomens (Mitra et al., 2012), was applied to the epithelial layer at an MOI of 1. The MOI was based on the number of epithelial cells in the top layer of the model. Models lacking myeloid cells were also activated with LPS or challenged with RSV to measure the activation of non-immune cells.

### 2.9 Flow cytometry

Models were digested in 2.2 mg/mL collagenase-D solution in PBS for 30 min with intermittent pipetting. Once digestion was complete, cells were rinsed with PBS and stained using ZombieGreen viability stain. See Supplementary Table S1 for antibody information. Staining for cell surface CD31, ICAM-1 and VCAM-1 was performed in 0.2% bovine serum albumin (BSA) and 0.09% sodium azide in PBS. Cells were analyzed on a BD Accuri™ C6 instrument.

Alternatively, to assess myeloid cell phenotypes, cells from six pooled models were stained with ZombieAqua viability dye (Biolegend) for 15 min at room temperature. Cells were then blocked with FcR block (Invitrogen) for 5 min before the addition of antibodies specific for myeloid cells (see table S1) for 15 min on ice. Cells were fixed with 4% paraformaldehyde for 10 min on ice. Cells were analyzed on an LSRII (BD Biosciences) or Aurora (Cytek) instrument.

### 2.10 Cytospin

Cell subsets were sorted based on staining of fluorescently labeled antibodies on an Aria (BD Biosciences). Cell subsets were loaded onto a cytospin™ four cytocentrifuge (Thermo Scientific) and spun down at 1000 RPM, medium acceleration for 5 min. Slides were fixed with methanol for 5 min and Wright-Giemsa stained. Slides were imaged on an Axiovert 200M (Zeiss) at 40x.

### 2.11 Multi-plex bead array

Media below the models were collected and stored at -80°C. Amounts of secreted IL-1β, IL-10, CCL2, TNFα, CXCL10, IFNβ, and IL-6 were quantified using a Luminex assay (R&D systems cat #LXSAHM) as per the manufacturer instructions. The Luminex assay was read on a BioPlex 200 (Bio-Rad).

### 2.12 RT-qPCR to detect RSV RNA

A portion of the single cell suspension obtained from the whole model was used to detect the presence of RSV F protein RNA by qPCR. RNA was isolated as per manufacturer specifications using an Arcturus^®^ PicoPure™ RNA isolation kit (Applied Biosystems). cDNA synthesis was performed using iScript gDNA Clear cDNA Synthesis Kit (Bio-Rad) and Heat ‘n run DNAse kit as per manufacturer specifications. Assay of RSV F gene expression was done with iTaq Universal Probes Supermix (Bio-Rad) with the primers/probe in Table S2 (Mentel et al., 2003). Gene expression was normalized to the housekeeping gene, *ACTB*.

### 2.13 WISH assay

Human IFN-responsive WISH cells were grown and plated as previously stated (Hua et al., 2006). Conditioned medium from 3D-HTLM wells was added to the WISH cells at a 3:1 ratio and incubated for 6 h. Then the medium was removed from the wells and spun down to collect any non-adherent cells. Trizol (Invitrogen) was added to the wells and collected with the non-adherent cell pellet. RNA was isolated as per manufacturer specifications with a DNeasy Mini Kit (Qiagen) followed by iScript gDNA clear cDNA synthesis kit (Bio-rad). IFN responsive genes (*MX1, IFIT1, PRKR*) (Supplementary Table S3) (Hua et al., 2006) were analyzed with iTaq Universal SYBR Green Supermix (Bio-Rad) on a Lightcycler 480 (Roche). Gene expression was normalized using 2^−ΔΔCT^ to *HPRT1* and the uninfected controls.

### 2.14 Dynamic qPCR arrays

The expression of 96 genes (Supplementary Table S4) was analyzed by qPCR using Dynamic Array 96.96 GE IFCs (Standard Biotools PN BMK-M-96.96) with Delta Gene assays according to manufacturer protocols. The qPCR was performed on a Biomark HD (Standard Biotools) using the Gene Expression thermal protocol. Data were analyzed by Fluidigm Real-Time PCR Analysis Software using the linear baseline correction, quality threshold 0.65 and Auto Ct Threshold Method. Gene expression was normalized using 2^−ΔΔCT^ to *HPRT1*, *GUSB1*, *GAPDH*, and *G6PD* housekeeping genes and the uninfected controls.

### 2.15 Statistical analysis

An experiment with each unique myeloid donor typically involved six model wells per condition. In the figure legends, “n” indicates the number of independent experiments. Flow cytometry data were analyzed with FlowJo 10. Graphpad Prism nine was used for statistical analysis. A two-tailed *t*-test, a one-way ANOVA with Tukey’s multiple comparisons test or a repeated measure two-way ANOVA with Sidak’s multiple comparisons test were used as appropriate. In all figures, * signifies *p* < 0.05, ** signifies *p* < 0.01, *** signifies *p* < 0.001, **** signifies *p* < 0.0001.

## 3 Results

### 3.1 3D-HTLM models contain three cell layers and show high TEER

**FIGURE 2:**
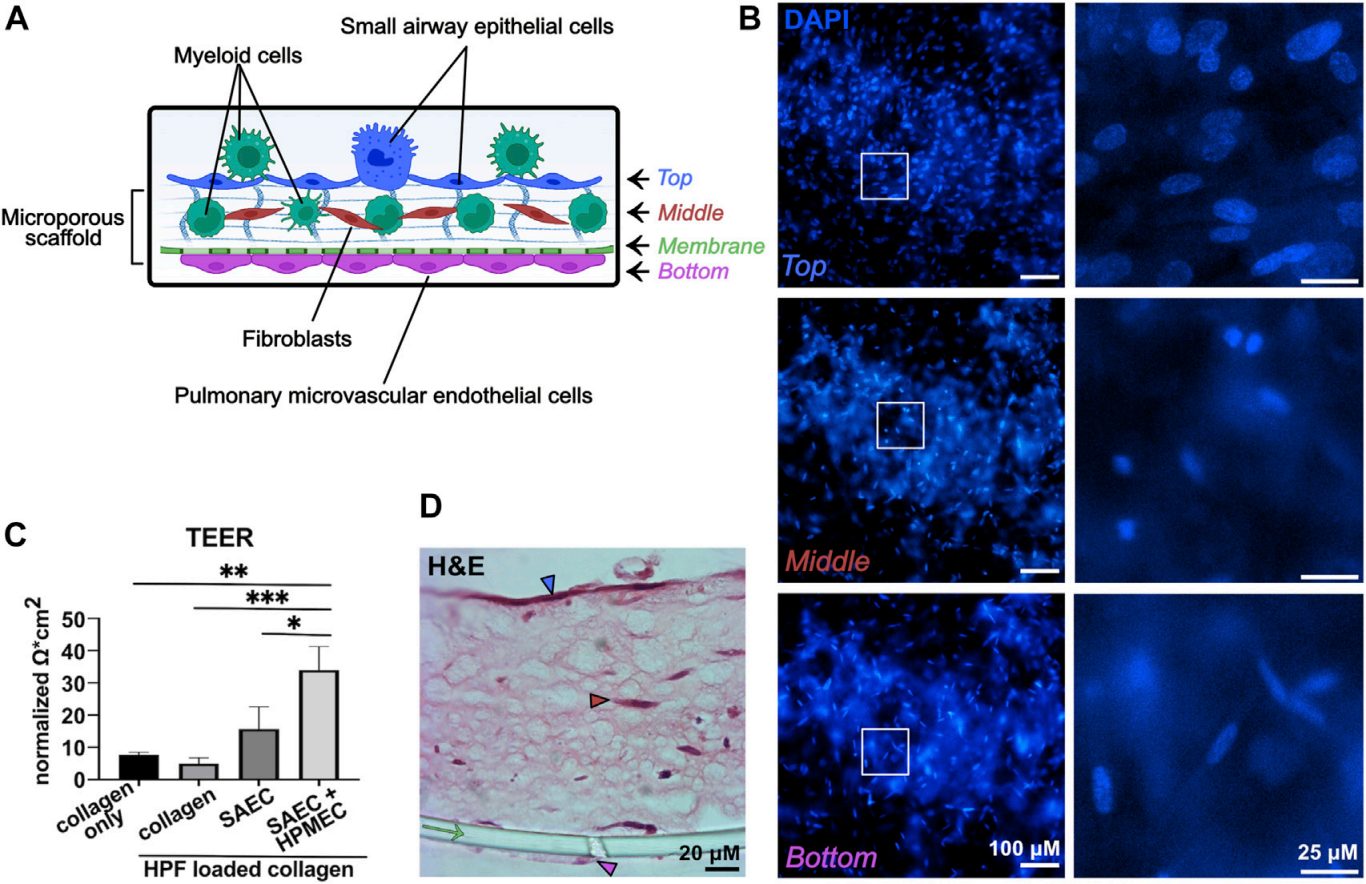
The 3D-HTLM contains three confluent cell layers and shows high TEER. **(A)** Graphical representation of the layers of the model. Graphic made with Biorender. **(B)** DAPI stained model imaged with fluorescence microscopy demonstrates the differences of nuclear morphology of each layer of the model. White boxes indicate the regions of interest, which are shown on the right at higher magnification. Each layer is indicated by the corresponding color in panel **(A)**. **(C)** TEER of individual and combined layers of 3D-HTLM on day 13. The data set was normalized to measurements obtained with the PET membrane and medium alone. One-way ANOVA with Tukey’s multiple comparisons test. * signifies *p* < 0.05, ** signifies *p* < 0.01, *** signifies *p* <0 .0001; n = 3. **(D)** H&E staining of paraffin embedded 3D-HTLM with myeloid cells added. The green arrow indicates the PET transwell membrane. The blue, red, and pink arrowheads correspond to the top, middle, and bottom layers indicated in panel **(A)**. Images shown are representative of 30 images taken from three wells.

Models were built stepwise with the three lung cell type donors remaining constant and myeloid cell donors varied (Figure 1). Models were stained and imaged in multiple ways to determine the confluency of the layers and the position of the myeloid cells in the model. The 3D-HTLM was fixed and stained on the transwell membrane for top-down imaging. Due to the thickness of the collagen layer, probing for specific cell types with top-down imaging did not reveal a definitive signal. However, with this method, the layers of the model (graphically depicted in Figure 2A) were distinguishable by the nuclear morphology. Using epifluorescence microscopy, nuclear images were taken of the top, middle, and bottom layers (Figure 2B) and this non-disruptive imaging technique demonstrated the confluency of the cells in each layer. The SAECs (Figure 2B, top images) on the top layer of the model had larger, oval nuclei compared with the fibroblasts within the collagen layer with smaller round nuclei (Figure 2B, middle images). The HPMECs on the bottom of the permeable membrane had long, thin nuclei (Figure 2B, bottom images). These differences in nuclear morphology between epithelial and endothelial cells of the lung also can be observed in human lung tissue sections (D'Agnillo et al., 2021).

To further demonstrate the confluency and formation of tight gap junctions of the SAECs and HPMECs, models were tested for resistance using TEER. There was a noticeable increase in resistance between the models containing an SAEC layer *versus* the collagen only and the collagen with HPFs. The complete model put together (SAEC + HPMEC with HPF in collagen) showed a significant increase in resistance compared with all other variations of the model without HPMECs (Figure 2C).

To visualize the layers of the model, 3D-HTLM models were fixed, and an agarose plug was added to the top and bottom of the transwell insert before paraffin embedding, transverse sectioning, and H&E staining (Figure 2D). With this sectioning method, all layers of the model were visible, with the SAECs denoted with a blue arrowhead, fibroblasts denoted with a red arrowhead, HPMECs denoted with a pink arrowhead, and the cell culture insert membrane denoted with a green arrow (Figure 2D). It should be noted that the H&E staining did not allow distinction of the myeloid cells within the model.

### 3.2 Blood myeloid cells added to the endothelial side of the 3D-HTLM migrate through all layers of the model

**FIGURE 3:**
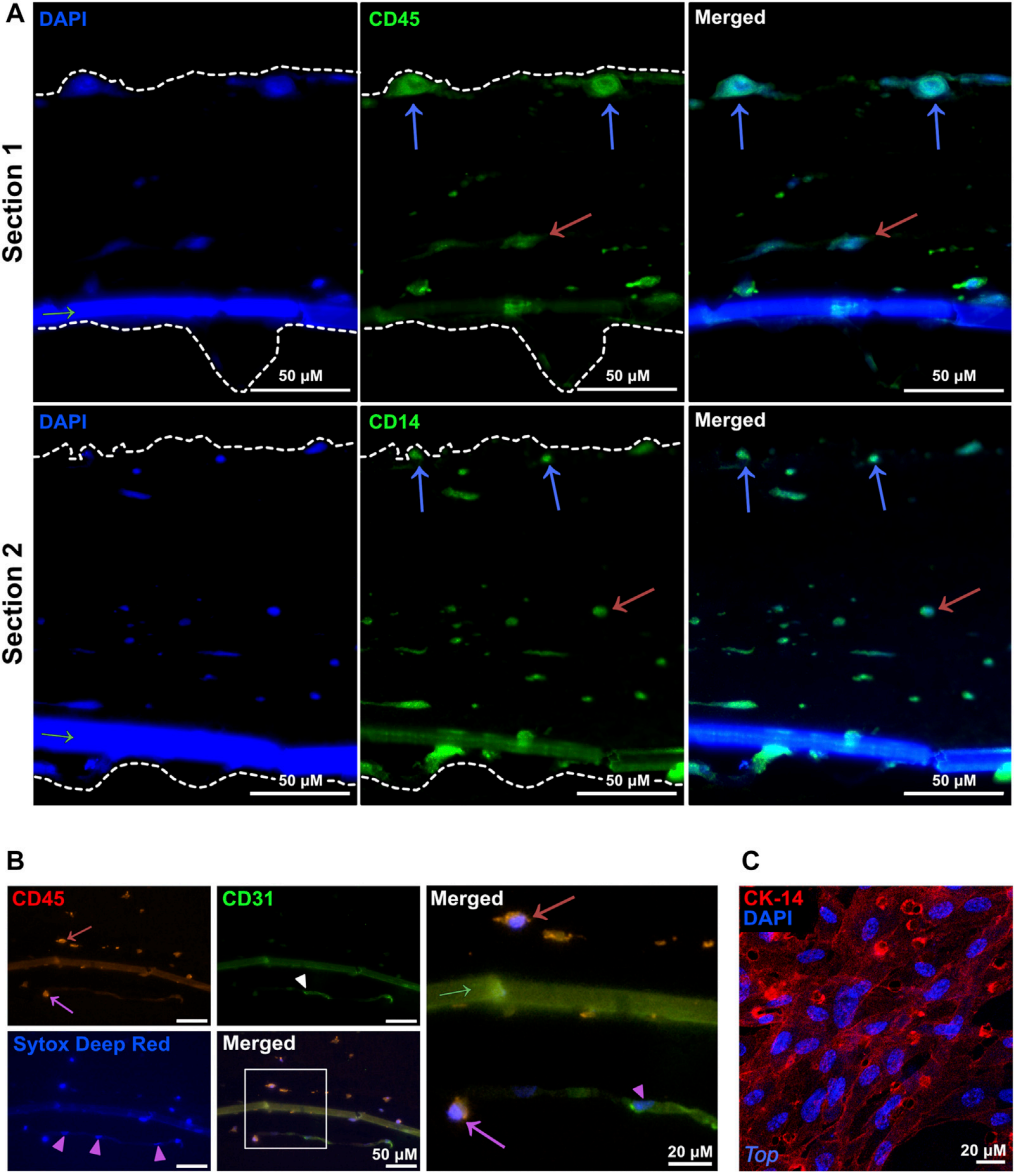
Myeloid cells migrate throughout all the layers of the 3D-HTLM. **(A,B)** Immunofluorescence of paraffin embedded and transverse sectioned 3D-HTLM. **(A)** Myeloid cells were detected with Abs for CD45 or CD14 (green) and nuclei were detected with DAPI. Blue and red arrows indicate myeloid cells within the top and middle layers of the model, respectively. Dotted lines outline the 3D-HTLM boundaries. Green arrow indicates the transwell membrane. **(B)** Abs for CD45 (red) detected myeloid cells and CD31 (green) detected HPMECs, and Sytox deep red was used to stain nuclei. A region of interest is outlined with a white box in the merged image, which is magnified in the panel to the right to highlight detail. Red and pink arrows indicate myeloid cells within the middle and bottom layers, respectively. Pink arrowheads indicate HPMEC nuclei. Green arrow indicates the transwell membrane. **(C)** The top SAEC layer of the 3D-HTLM was removed and probed with an Ab for CK-14 (R-PE) to locate SAECs. Nuclei were counterstained with DAPI. Images shown are representative of 40 images taken from three wells.

To characterize the cells within the layers 4 days after addition of myeloid cells to the 3D-HTLM, we performed immunofluorescence staining on the paraffin embedded sections. CD45^+^ myeloid cells added to the endothelial side of the model were found to reside at the top of the model at the epithelial layer (Figure 3A, blue arrows), throughout the collagen layer (Figures 3A, B, red arrows), and within the endothelial layer (Figure 3B, pink arrow). The boundaries of the model, denoted with white-dashed lines, were identified by increasing the fluorescence to visualize where the collagen layer ends (Figure 3A). The nuclei of the CD31^+^ HPMECs (Figure 3B, pink arrowheads) show similar morphology to the bottom layer of the model imaged in Figure 2B, bottom panel. This method of sample preparation often led to the loss of the epithelial layer. To characterize the SAEC layer, the 3D-HTLM was fixed and the SAEC layer was removed from the PET membrane and probed for CK-14, an epithelial cell marker. Figure 3C shows a confluent layer of cells with expression of CK-14. In addition, the nuclei of the cells expressing CK-14 have morphology similar to the top layer nuclei imaged in Figure 2B, top panel. These data demonstrate the confluency of the SAECs and HPMECs of the 3D-HTLM and show that the myeloid cells are present in each layer of the model after 4 days.

### 3.3 Blood myeloid cells added to the 3D-HTLM acquire a tissue resident phenotype

**FIGURE 4:**
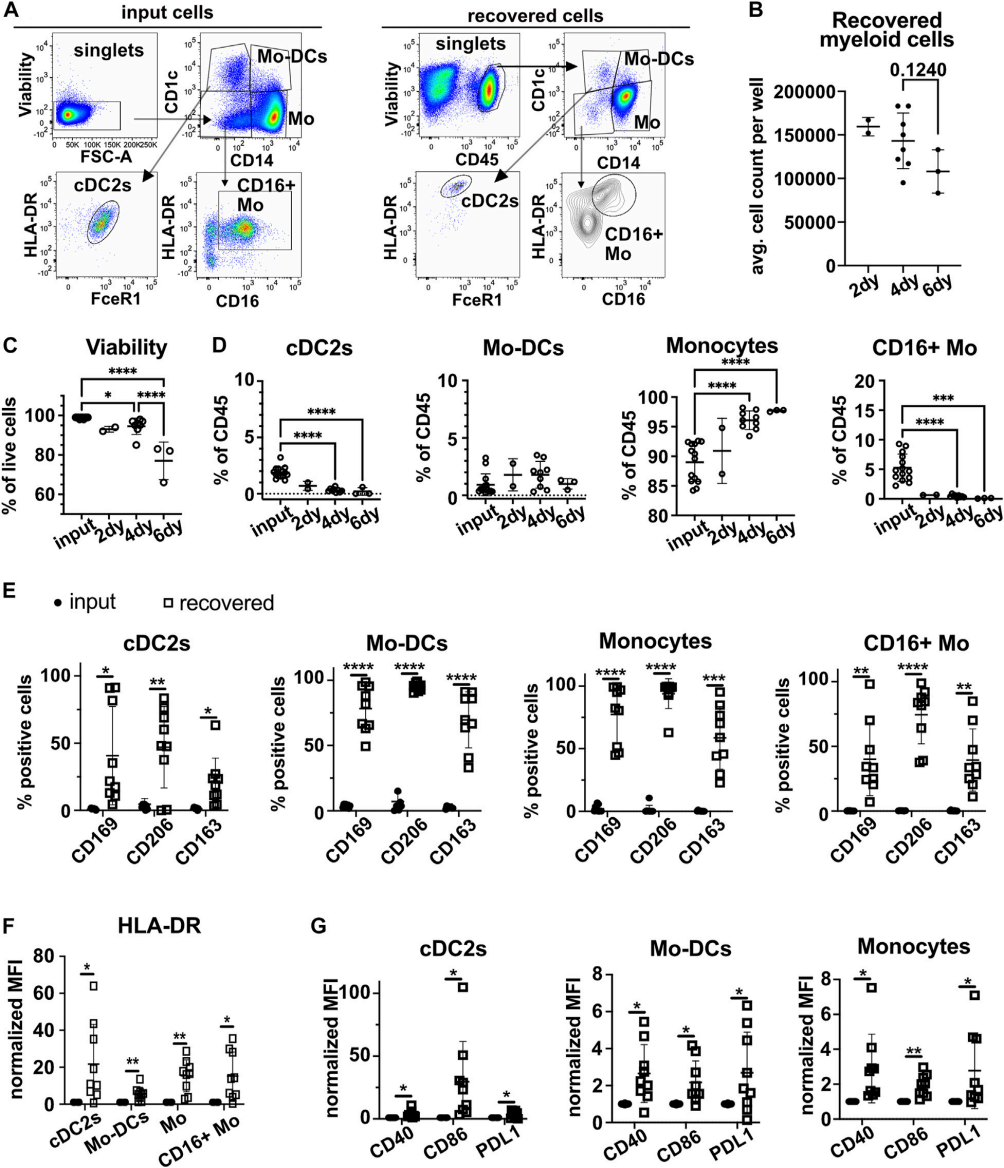
Myeloid cells added to the 3D-HTLM acquire a tissue resident phenotype. Myeloid cells added to the 3D-HTLM include small subsets of cDC2s, Mo-DCs, classical CD14^+^ Mo, and non-classical CD16^+^ Mo as identified by CD14, CD16, and CD1c expression by flow cytometry. **(A)** Representative flow cytometry gating strategy of input (left) cells and recovered (right) cells. **(B)** The number of input myeloid cells remained constant for all wells (2.5 × 10^5^/well). Average number of live myeloid cells recovered per well at different time points. **(C)** Viability of myeloid cells from the model at different time points. **(D)** Percentage of live (input) or CD45^+^/live (recovered) cells after 2 (n = 2), 4 (n = 9), or 6 (n = 3) days in the 3D-HTLM. n indicates the number of models with unique myeloid donors tested; symbols represent data from unique models. One-way ANOVA with Tukey’s multiple comparisons test between input, day 4, and day 6. Day 2 was excluded from statistics due to low n. * represents statistical significance from the input cells. **(E)** Percentage of cells of each myeloid subset that are expressing lung tissue resident markers before and after recovery from the 3D-HTLM after 4 days. **(F,G)** Normalized MFI of **(F)** HLA-DR and **(G)** costimulatory markers of input and recovered cells from the 3D-HTLM after 4 days. **(E–G)** Paired two-tailed *t*-test of each marker between input and recovered of each subset (n = 9).

To determine how myeloid cells adapt to the 3D-HTLM environment, models were collagenase digested to a single cell suspension and assayed by flow cytometry using specific antibodies to define myeloid subsets. The majority of myeloid cells added to the 3D-HTLM were CD14^+^ Mo (CD1c^−^CD14^+^), but the input cells included minor subsets of cDC2s (CD1c^+^CD14^−^FcεR1^+^), Mo-DCs (CD1c^+^CD14^+^), CD16^+^ Mo (CD1c^−^CD14^−^CD16^+^) (Figure 4A), and cDC1s (CD1c^−^CD14^−^CD16^−^CD141^hi^) (*data not shown*). The average number of myeloid cells recovered per model did not significantly change after 2, 4, or 6 days in the model (Figure 4B). However, the viability of the myeloid cells in the model did decrease over time (Figure 4C). After 2, 4, and 6 days, the input myeloid cell subsets were recovered from the 3D-HTLM (Figures 4A,D), except for cDC1s, which were not recovered at any of the time points (*data not shown*). Additionally, the proportion of recovered cDC2s and CD16^+^ Mo was diminished compared with input proportions over time (Figure 4D). To optimize the presence of the minor cell subsets, all further experiments were conducted with myeloid cells added to the 3D-HTLM for a total of 4 days.

Myeloid cells recovered from the 3D-HTLM showed a significant increase in the percentage of cells that expressed the tissue resident markers CD169, CD206, and CD163 compared with the input cells within each subset (Figure 4E). All myeloid subsets had a significant increase in the expression of HLA-DR compared with input cells (Figure 4F). Mo, Mo-DCs, and cDC2s had increased expression of activation markers CD40, CD86, and PDL1 (Figure 4G). These data demonstrate that the tissue environment provided by the 3D-HTLM supports the differentiation of human blood myeloid cells into cells with a lung tissue resident phenotype.

We next determined if upregulation of these tissue resident markers on blood myeloid cells required their residence in the 3D-HTLM or would occur in response to soluble factors produced in the 3D-HTLM environment. We exposed myeloid cells to the cytokines originally added to the model (GM-CSF, FLT3L, SCF) or to conditioned medium isolated from the 3D-HTLM lacking myeloid cells combined with the cytokines (GM-CSF, FLT3L, SCF) in standard tissue culture wells. We saw similar proportions of cells expressing CD206 and CD163 in all subsets after culture in medium alone or in 3D-HTLM conditioned medium (Supplementary Figure S1). However, the proportion of Mo and Mo-DCs with CD169 expression was lower under these tissue culture conditions compared with myeloid cells recovered after residence in the 3D-HTLM (Supplementary Figure S1). This finding suggests that proximity to other lung resident cell types is needed for blood myeloid cells to fully acquire tissue resident phenotypes.

### 3.4 A subset of cells characterized as alveolar macrophages was recovered from the 3D-HTLM

**FIGURE 5:**
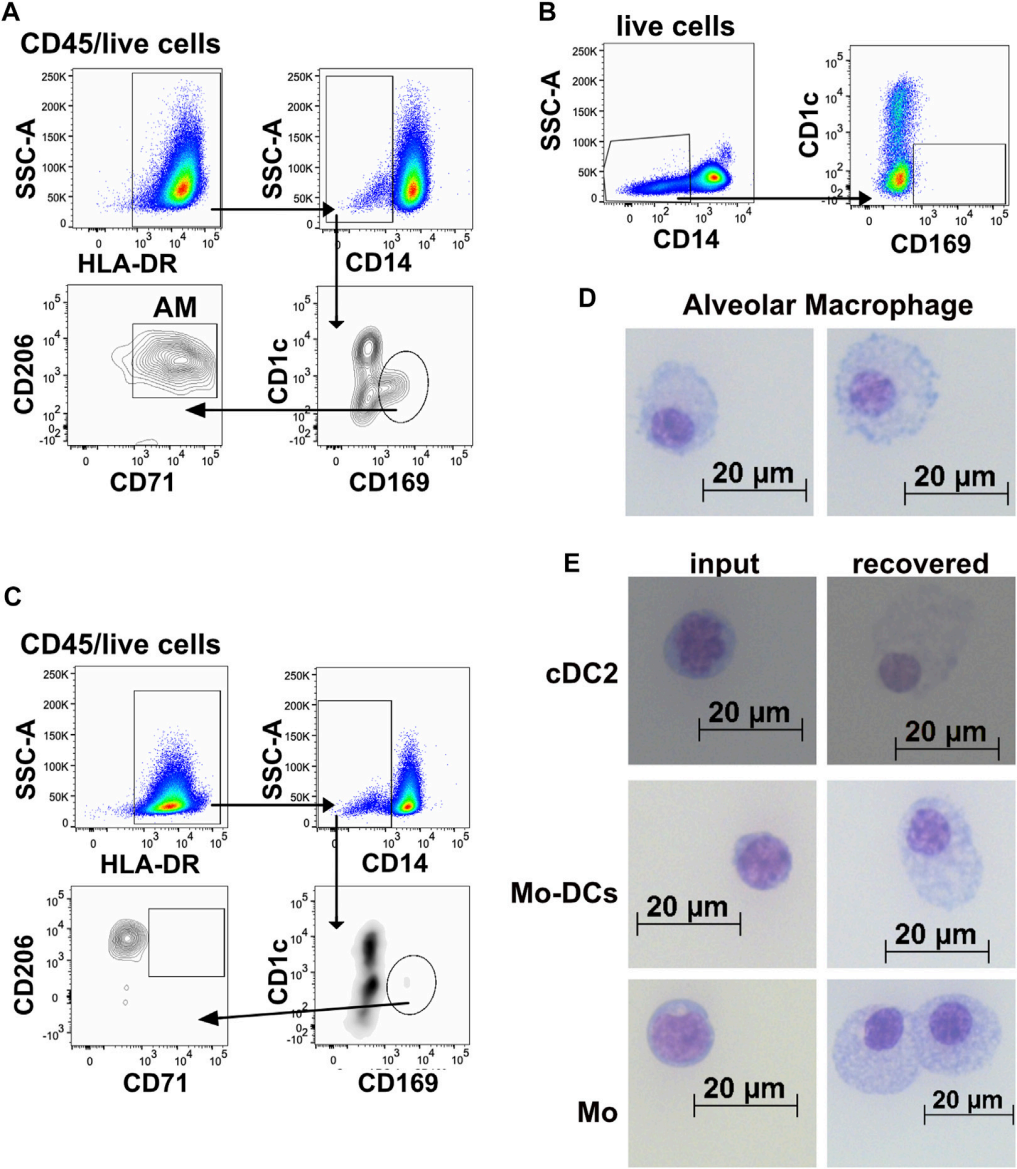
Cells characterized as alveolar macrophages were recovered from the 3D-HTLM. **(A)** Representative flow cytometry gating strategy of myeloid cells identified as alveolar macrophages (AM) recovered from 3D-HTLM. **(B)** Gating demonstrating the absence of AM in the input cells and **(C)** in monocytes cultured alone with GM-CSF, SCF, and FLT3. **(D,E)** Input and recovered cells from the 3D-HTLM were sorted, adhered to slides using a cytospin, and Giemsa stained. **(D)** Images of cells distinguished as alveolar macrophages. **(E)** Input (left) and recovered (right) cells from each of the myeloid subsets introduced to the 3D-HTLM. Images shown are representative of 140 images taken with cells sorted from 24 pooled wells obtained from three separate experiments.

In addition to tissue resident markers CD206 and CD169, alveolar macrophages (AM) express CD71 and can be distinguished from interstitial macrophages (IM) based on the absence of CD169 and CD71 on IM (Yu et al., 2016; Patel et al., 2017; Evren et al., 2021). We sought to assess whether Mo added to the 3D-HTLM could differentiate into cells with features of AM. Cells with characteristics of AM were recovered from the 3D-HTLM and were gated as CD1c^−^CD14^lo/−^HLA-DR^+^CD169^+^CD206^+^CD71^+^ (Figure 5A). This subset of cells was not found within the input cells (Figure 5B) nor when Mo were cultured alone on plastic wells in the presence of GM-CSF, SCF, and FLT3L for 4 days (Figure 5C). To evaluate the morphologic changes of myeloid cells after recovery from the 3D-HTLM, input and recovered cells were sorted using flow cytometry, spun onto slides by a cytospin centrifuge and Giemsa stained. AMs recovered from the 3D-HTLM had marked differences from input Mo (Figures 5D,E) and had morphological characteristics similar to primary AMs recovered from human BAL and digested lung tissue (Yu et al., 2016; Patel et al., 2017), such as a small nucleus to cytoplasm ratio, “lacy” cytoplasm, and a ruffled cellular membrane. Changes in the morphology of cells from the other recovered subsets were also observed (Figure 5E). The recovered Mo subset (CD1c^−^CD14^+^CD16^+/−^) showed characteristics of macrophages with a small nucleus to cytoplasm ratio compared with input Mo (Figure 5E). These findings further support the differentiation of input Mo into tissue resident cells, including cells characterized as AM.

### 3.5 Bacterial lipopolysaccharide stimulation of the 3D-HTLM leads to increased ICAM-1 expression on CD31^+^ HPMECs and increased secretion of IL10, TNF⍺, and IL-6 from the myeloid cells

**FIGURE 6:**
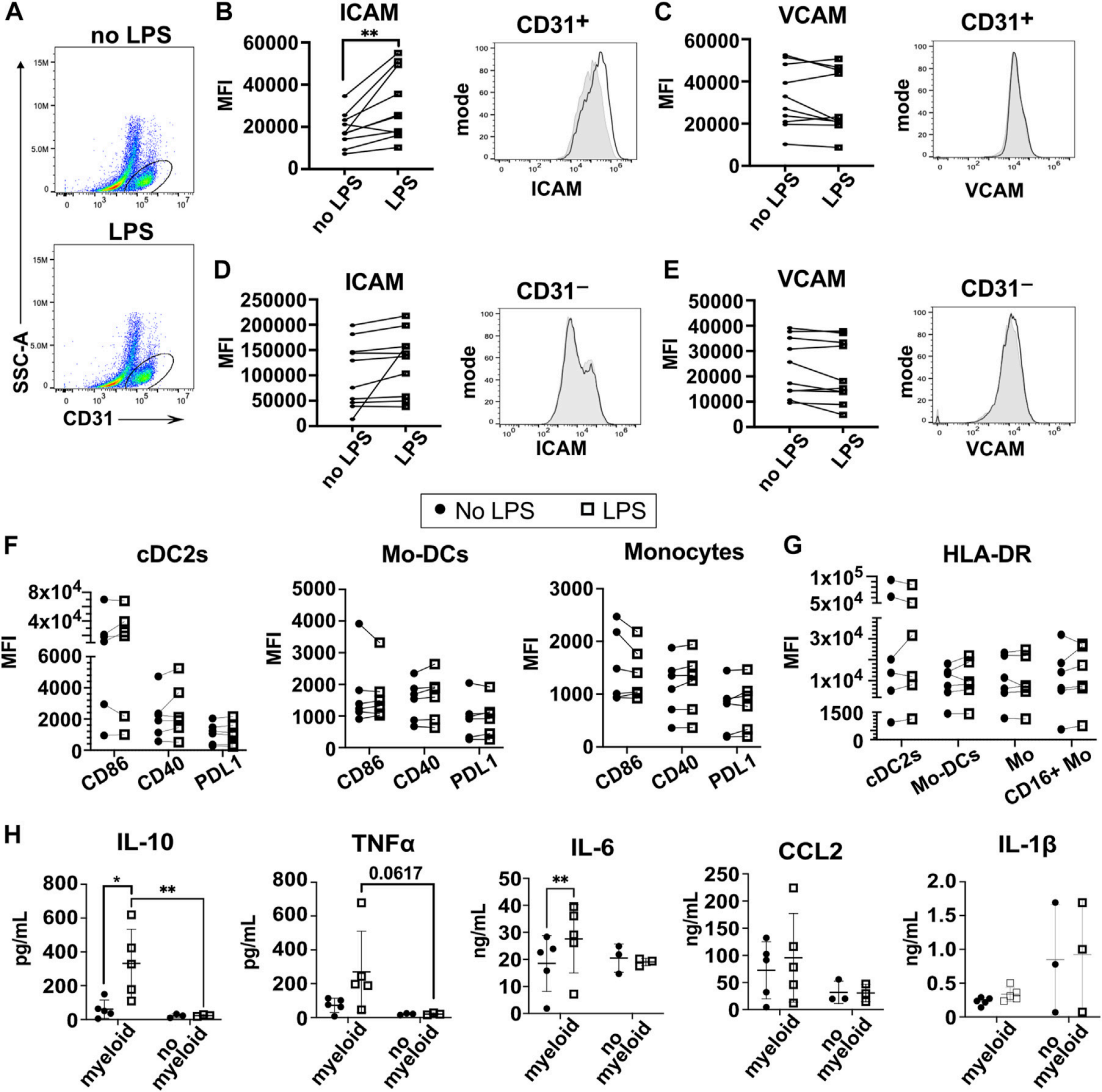
LPS stimulation of the 3D-HTLM leads to increased ICAM-1 expression on CD31^+^ HPMECs and increased secretion of IL-10, TNF⍺, and IL-6 dependent on myeloid cells. **(A)** FACs gating strategy to identify CD31^+^ endothelial cells with or without LPS stimulation. **(B,C)** Mean fluorescence intensity (MFI) and representative FACs histogram of **(B)** ICAM and **(C)** VCAM with LPS stimulation on CD31^+^ endothelial cells. Paired two-tailed *t*-test (n = 10) Grey, filled line = no LPS; black, unfilled line = +LPS. **(D,E)** MFI and representative FACs histogram of **(D)** ICAM and **(E)** VCAM with LPS stimulation on CD31^−^ cells. Paired two-tailed *t*-test (n = 10) Grey, filled line = no LPS; black, unfilled line = +LPS. **(F,G)** MFI of **(F)** the activation markers CD86, CD40, and PDL1 and **(G)** HLA-DR of recovered myeloid subsets with or without stimulation of LPS. Paired two-tailed *t*-test (n = 6). **(H)** Multi-plex bead array analysis of the supernatant from 3D-HTLM with or without the addition of myeloid cells and stimulated with LPS. * represents *p* < 0.05, ** represents *p* < 0.01. Repeated measure two-way ANOVA with Sidak’s multiple comparisons test.

We next characterized the response of the 3D-HTLM to an innate immune system stimulus, the TLR4 agonist bacterial LPS. LPS was reported to increase ICAM-1 on both endothelial and type I alveolar epithelial cells (Sawa et al., 2008; Cho et al., 2016). LPS was added to the epithelial side of the model (Figure 1) for the last 24 h before harvest. The response of HPMECs to LPS activation was assessed by expression of ICAM-1 and VCAM-1 using flow cytometry (Figures 6A–C). HPMECs were identified by the expression of CD31 (Figure 6A). CD31^+^ HPMECs increased expression of surface ICAM-1 after LPS stimulation, but no notable change in VCAM-1 was found (Figures 6B,C). We did not detect a change in the expression of ICAM-1 or VCAM-1 in the CD31^−^ cells after LPS stimulation (Figures 6D,E). These data demonstrate a functional response of the HPMECs to LPS stimulation in the 3D-HTLM.

Analyses of the myeloid cells recovered from the 3D-HTLM after LPS exposure showed that some myeloid cell donors increased co-stimulatory markers (CD40, CD86, PDL1) and HLA-DR expression, but combined analyses of these markers on all donors did not show statistically significant differences between LPS stimulated and unstimulated myeloid cells (Figures 6F,G).

To further characterize the response of the myeloid cells to LPS stimulation of the 3D-HTLM, the medium on the endothelial side of the model was analyzed by multi-plex bead array to detect secreted inflammatory cytokines. Media were collected from the 3D-HTLM with and without added myeloid cells and/or LPS stimulation. The LPS stimulated 3D-HTLM containing myeloid cells showed increased secretion of IL-10 and IL-6 compared with the 3D-HTLM lacking myeloid cells or the 3D-HTLM containing myeloid cells without LPS stimulation (Figure 6H). TNFα secretion was increased in each individual myeloid donor in the 3D-HTLM stimulated with LPS; however, the data lacked statistical significance due to wide variation between individual donor responses (Figure 6H). The secretion of CCL2 and IL-1β was independent of myeloid cells in the 3D-HTLM and LPS stimulation (Figure 6H). These data demonstrate the functionality of the different cell types after stimulation of the model with LPS.

### 3.6 3D-HTLM models challenged with RSV showed increased expression of genes involved in viral sensing, macrophage activation, and IFN signaling

**FIGURE 7:**
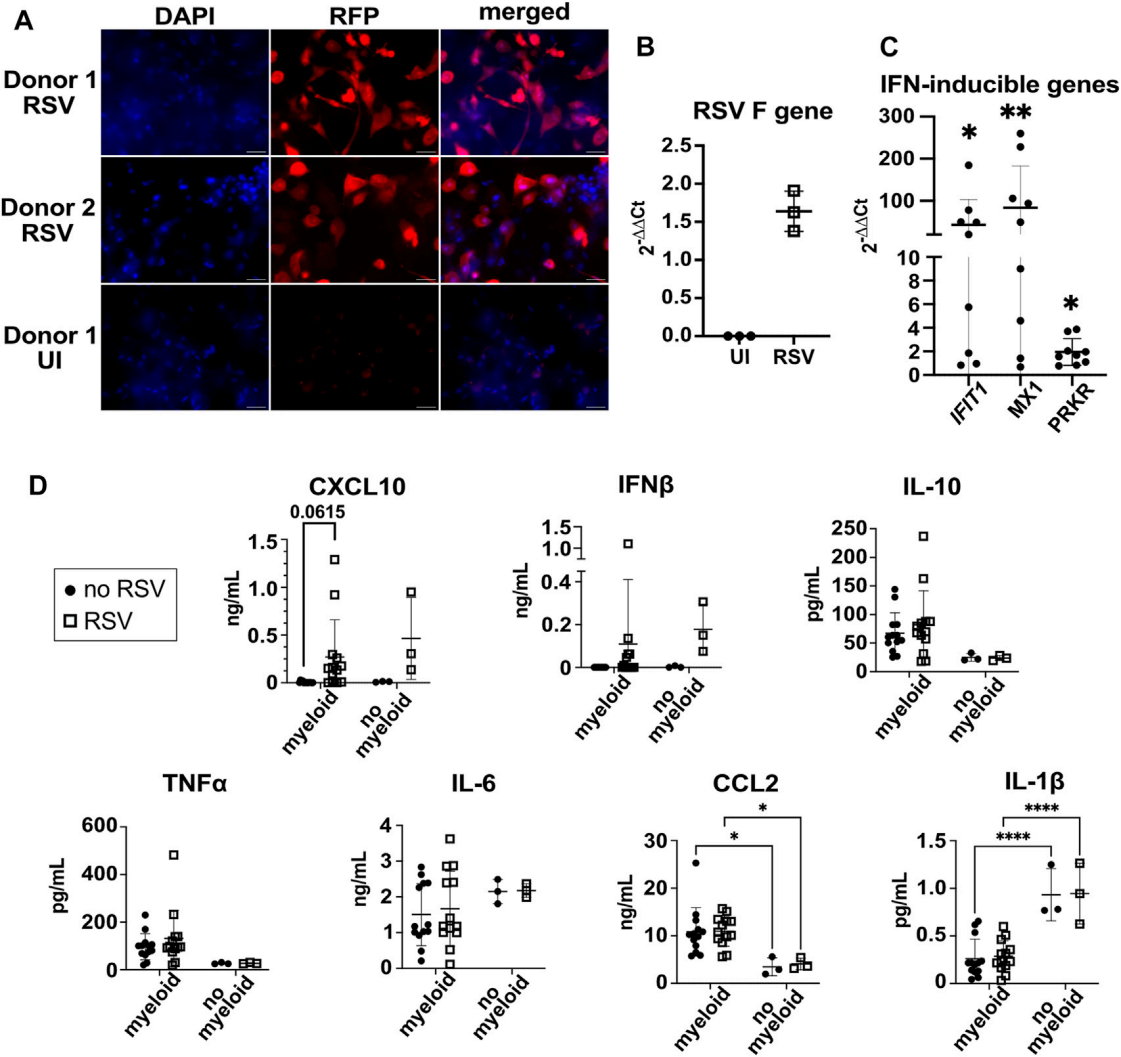
3D-HTLM models challenged with RSV had increased production of type I IFN and expression of IFN-response genes. **(A)** Fluorescence microscopy of whole mount models challenged with RFP-RSV. Images shown are representative of 10 images taken from two wells. **(B)** RT-qPCR analysis of the gene encoding the RSV F protein 24 h after challenge. An aliquot of the whole model digest was used for RNA isolation. **(C)** Supernatant from RSV-challenged or uninfected models were added to a monolayer of WISH cells for 6 h. IFN-inducible genes, *IFIT1*, *MX1*, and *PRKR* RNA was measured by RT-qPCR. * indicates significance from uninfected control supernatants. Wilcoxon paired two-tailed *t*-test. **(D)** Multi-plex bead array analysis of the supernatant from 3D-HTLM with or without the addition of myeloid cells and challenged with RSV. * represents *p* < 0.05, ** represents *p* < 0.01, **** represents *p* < 0.0001. Repeated measure two-way ANOVA with Sidak’s multiple comparisons test.

The epithelial surface of model wells was infected with RSV at MOI = 1 (based on epithelial cell numbers) for 24 h. Fluorescence microscopy images of the 3D-HTLM show that the majority of the cells within the top layer of the model were infected with RFP-RSV (Figure 7A). However, we did not observe cytopathic effects after 24 h of RSV challenge. RT-qPCR for the gene encoding the RSV F protein showed the presence of RSV RNA in the complete infected models 24 h post challenge (Figure 7B).

To test for secretion of type I IFN, we added supernatant from RSV challenged models to IFN-responsive WISH cells and analyzed the expression of IFN response genes by qPCR. We found a significant increase in the expression of *IFIT1*, *MX1*, and *PRKR* in WISH cells upon exposure to supernatants from RSV-challenged models (Figure 7C), suggesting type I IFN production by the model. The supernatant on the endothelial side of the model was collected and used for a multiplex bead array to determine cytokine secretion. Increased secretion of CXCL10 was observed in most of the samples, yet the data did not reach statistical significance due to the variation between individual myeloid cell donor responses (Figure 7D). Despite the evidence for type I IFN induction obtained with the WISH assay, RSV challenge did not lead to a significant accumulation of IFNβ secretion after RSV challenge (Figure 7D). The production of TNF⍺, IL-10, and IL-6 was independent of myeloid cells in the model and RSV challenge (Figure 7D). The addition of myeloid cells to the model led to increased secretion of CCL2 and decreased secretion of IL-1β independent of RSV challenge (Figure 7D).

**FIGURE 8:**
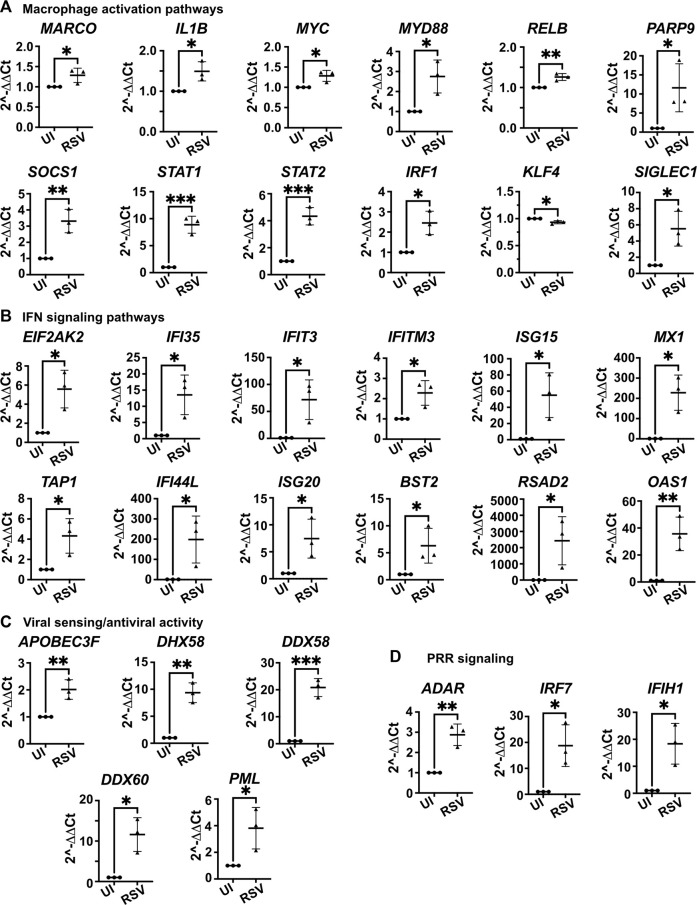
Whole model dynamic qPCR array shows increased expression of genes associated with macrophage activation, IFN signaling, viral sensing and antiviral activity, and PRR signaling after challenge with RSV. **(A)** Genes associated with macrophage activation pathways. **(B)** Genes associated with IFN signaling pathways. **(C)** Genes associated with viral sensing and antiviral activity. **(D)** Genes associated with PRR signaling. IPA used to determine pathways of gene association. * represents *p* < 0.05, ** represents *p* < 0.01, *** represents *p* < 0.001. n = 3 myeloid donors. Unpaired two-tailed *t*-test.

To further characterize the response of the 3D-HTLM to RSV challenge, RNA isolated from the whole models independently built from three myeloid donors was used in a dynamic RT-qPCR array of 96 genes (Figure 8). Patterns of gene expression were determined with Ingenuity Pathway Analysis (IPA). Genes associated with macrophage activation pathways (classical and non-classical), IFN signaling pathways, viral sensing and anti-viral activity, and pattern recognition receptor (PRR) signaling pathways showed increased expression after RSV challenge of the model (Figures 8A–D). These data were replicated through the use of one myeloid donor in models built on separate days, and the duplicate models showed similar expression profiles of most genes (Supplementary Figure S2). Comparison of patterns of gene expression among three unique myeloid cell donors shows individual variation in responses to RSV challenge in the model, although the gene expression trends were maintained (Supplementary Figure S3). These data demonstrate the robust host antiviral response and the activation of myeloid cells within the 3D-HTLM upon RSV challenge.

## 4 Discussion

Here we present a 3D human tissue-engineered lung model (3D-HTLM) that mimics the lower airway cellular environment and can be used to assess human innate immune responses to pathogens. The model was built stepwise using primary human cells, resulting in a layer of small airway epithelial cells grown at air-liquid interface above a layer of fibroblasts embedded in a collagen matrix, which is resting on a permeable membrane underlaid with pulmonary microvascular endothelial cells. The placement of epithelial and endothelial layers in the model led to increased barrier integrity of the complete 3D-HTLM, a property also observed in colonic and alveolar-capillary co-culture models with epithelial and endothelial layers (Janga et al., 2018; Bian et al., 2020; Kaeokhamloed et al., 2021). Blood myeloid cells (containing monocytes and DCs) added to the endothelial layer acquired properties of lung tissue resident cells, migrated throughout the model, and occupied spaces that enabled interactions with the other cell types in each layer of the model. Thus, this complete model can be used to provide important information about human innate immune responses to respiratory pathogens in the context of a physiological multi-cellular environment. Importantly, the stepwise construction of the model will allow future experiments to selectively omit or modify particular cell types to determine their contribution to innate responses assessed in specific cell types or at the level of the entire model. In addition, we demonstrated the feasibility of using cell sorting to isolate myeloid cell types from the built model in order to determine cellular and molecular phenotypes shaped by the 3D tissue environment.

Another strength of the model is the use of primary human cells since their functional responses will most accurately reflect those of intact human tissue. Over time, the model will provide information about donor variation in responses. While we used a single adult donor for each of the epithelial and endothelial cell and fibroblast populations, we varied the myeloid cell donors. Future experiments will vary the age and gender of each cell type to increase our understanding of how these biological variables impact innate immune responses.

Characterization of the myeloid cells within the model revealed the Mo subset was found at higher proportions when cultured for 6 days compared with 2 and 4 days, whereas the CD16^+^ Mo and cDC2s decreased in proportion. These data were expected as DCs have a short half-life (Guilliams and van de Laar, 2015). However, the fate of the CD16^+^ Mo within the model is less certain. In a humanized mouse model, CD16^+^ monocytes were shown to differentiate into intravascular macrophages that reside along the blood vessels within the lung (Evren et al., 2021). Immunofluorescence shows CD45^+^ myeloid cells within the HPMEC layer of the 3D-HTLM, but it is unclear if the 3D-HTLM supports the differentiation of this specialized macrophage subset. In addition, the increased proportion of Mo after 6 days in the model suggests that the Mo may be differentiating into macrophages, since macrophages have a much longer half-life than monocytes (Parihar et al., 2010). This finding is also supported by our observation that Mo acquire morphological features of lung resident macrophages including a smaller nuclear to cytoplasmic ratio (Figure 5E).

Analysis of the cellular phenotypes of the myeloid cells recovered from the 3D-HTLM shows increased proportions of cells expressing the tissue resident markers, CD206, CD169, and CD163 in the subsets analyzed. These findings corroborate previous literature demonstrating that tissue resident macrophage and DC populations express higher levels of CD206 compared with circulating Mo and DC populations (Zizzo et al., 2012; Baharom et al., 2016; Ruiz-Alcaraz et al., 2018). Additionally, in human lung tissue, resident DCs and peritoneal macrophages have higher expression of HLA-DR, CD86, and CD40 compared with blood DCs and monocytes, respectively (Baharom et al., 2016; Desch et al., 2016; Patel et al., 2017; Ruiz-Alcaraz et al., 2018). These findings are similar to our observation that the recovered myeloid cell subsets from the 3D-HTLM show increased expression of HLA-DR, CD86, and CD40 compared with input cells.

After recovery from the 3D-HTLM, we identified a minor subset of cells with characteristics of AM based on surface expression and morphology. *In vitro* studies of monocyte-derived macrophages (MDM) have revealed many useful insights into the plasticity of macrophages, although the assay conditions are not ideal for the differentiation of AM. GM-CSF is essential for AM development, yet Mo cultured with GM-CSF, to induce differentiation of MDM, fail to express all of the markers that identify AM *in vivo* (Rey-Giraud et al., 2012; Zizzo et al., 2012). We also found that after culture of Mo and Mo-DCs with 3D-HTLM conditioned media, the proportion of cells expressing CD169 did not reach the levels seen in cells recovered from the 3D-HTLM (Supplementary Figure S1). These data suggest that the expression of CD169 may be upregulated through contact-dependent mechanisms that are not required for acquisition of the tissue resident markers CD206 and CD163. Additional investigation of the functionality and transcriptome of the cells recovered from the 3D-HTLM would provide more insight into this question. Multiple studies in mice have shown that circulating monocytes recruited by CCL2 can reconstitute a depleted AM niche (van de Laar et al., 2016; Evren et al., 2021; Venosa et al., 2021). We have shown that CCL2 is secreted by cells within the 3D-HTLM independent of stimulation or the presence of myeloid cells (Figures 6H, 7D). This suggests that there are additional yet unknown factors within the lung environment that contribute to the differentiation of blood Mo into AM. Given that AM reside within the alveolar space and are important for clearance of pathogens (Kolli et al., 2014; Huang et al., 2019), a model that can support AM is well equipped to study respiratory bacterial and viral infection.

To understand the functional capacity of the model and added myeloid cells, the 3D-HTLM was challenged with bacterial LPS and RSV. Myeloid cells recovered from the 3D-HTLM after stimulation with LPS did not increase expression of co-stimulatory markers (Figures 6F,G). This finding is similar to reports of human BAL cells that were stimulated with heat-killed bacteria and showed no changes in the expression of CD86 (Patel et al., 2017), suggesting that lung resident myeloid cells are partially activated in response to tissue signals. Thus, the myeloid cells within the 3D-HTLM may be in a poised activation state, yet capable of responding to LPS stimulation by producing cytokines. Indeed, the LPS-stimulated 3D-HTLM showed a myeloid cell-dependent increase in the secretion of IL-10, IL-6, and TNFα (Figure 6H). This is consistent with previous *ex vivo* studies from human lung that demonstrated increased TNFα and IL-10 secretion upon LPS stimulation, with macrophages as the main producers of TNFα (Hackett et al., 2008). Taken together, our data show that the myeloid and endothelial cells within the 3D-HTLM rapidly respond to exogenous challenge by an innate stimulus.

The importance of type I IFN signaling in response to RSV infection has been demonstrated by the increased morbidity and higher viral loads in type I IFN receptor (IFNAR) knockout mice compared with wild type mice (Goritzka et al., 2014). The levels of IFNβ and CXCL10 secretion after RSV challenge of the model were unexpectedly low (Figure 7D), although we did detect type I IFN activity in 3D-HTLM conditioned medium using sensitive reporter WISH cells. This may be due to RSV proteins that facilitate evasion of the IFN response in the host (Bossert et al., 2003; Spann et al., 2004) and the fact that all of the cells within the 3D-HTLM have receptors for IFNAR (Navarro et al., 1996) and may quickly consume the type I IFN released from cells in the model. In spite of the low levels of secreted IFNβ, our PCR data provide evidence of an IFN response, with the increased expression of genes associated with IFN signaling, DC and macrophage activation, and viral sensing and PRR signaling pathways (Figure 8) demonstrating that the cells within the 3D-HTLM are responding to RSV challenge. These results corroborate previous findings that children admitted to the hospital with severe RSV infection show activation of IFN signaling pathways (Bucasas et al., 2013), suggesting that the 3D-HTLM can recapitulate the response seen *in vivo*. Interestingly, incorporation of myeloid cells into the model led to reduced production of IL-1β, suggesting alternate regulation of IL-1β synthesis or IL-1 antagonists in myeloid cells (Chan and Schroder, 2020) and revealing new insights into patterns of cytokine production in the context of multiple pulmonary cell types.

We here have demonstrated a 3D lung model that facilitates the differentiation of cells with characteristics of alveolar macrophages and other tissue resident cells from enriched monocytes. These myeloid cells are phenotypically and morphologically distinct from input cells and have the capacity to respond to LPS and RSV stimulation. In addition, cocultured epithelial and endothelial cell layers with a fibroblast-loaded collagen scaffold demonstrate barrier integrity and permit positioning of myeloid cells in each layer, thus resembling lung tissue *in vivo*. This model will provide a platform for future insights into human innate immune responses to respiratory infections.

## Data Availability

The datasets presented in this study can be found in online repositories. The names of the repository/repositories and accession number(s) can be found in the article/Supplementary Material.
