# Supplementary figures and images for: Blood myeloid cells differentiate to lung resident cells and respond to pathogen stimuli in a 3D human tissue-engineered lung model
Source: Front Bioeng Biotechnol. 2023 Jul 7;11:1212230. doi: 10.3389/fbioe.2023.1212230 (PMC10361305; doi:10.3389/fbioe.2023.1212230)

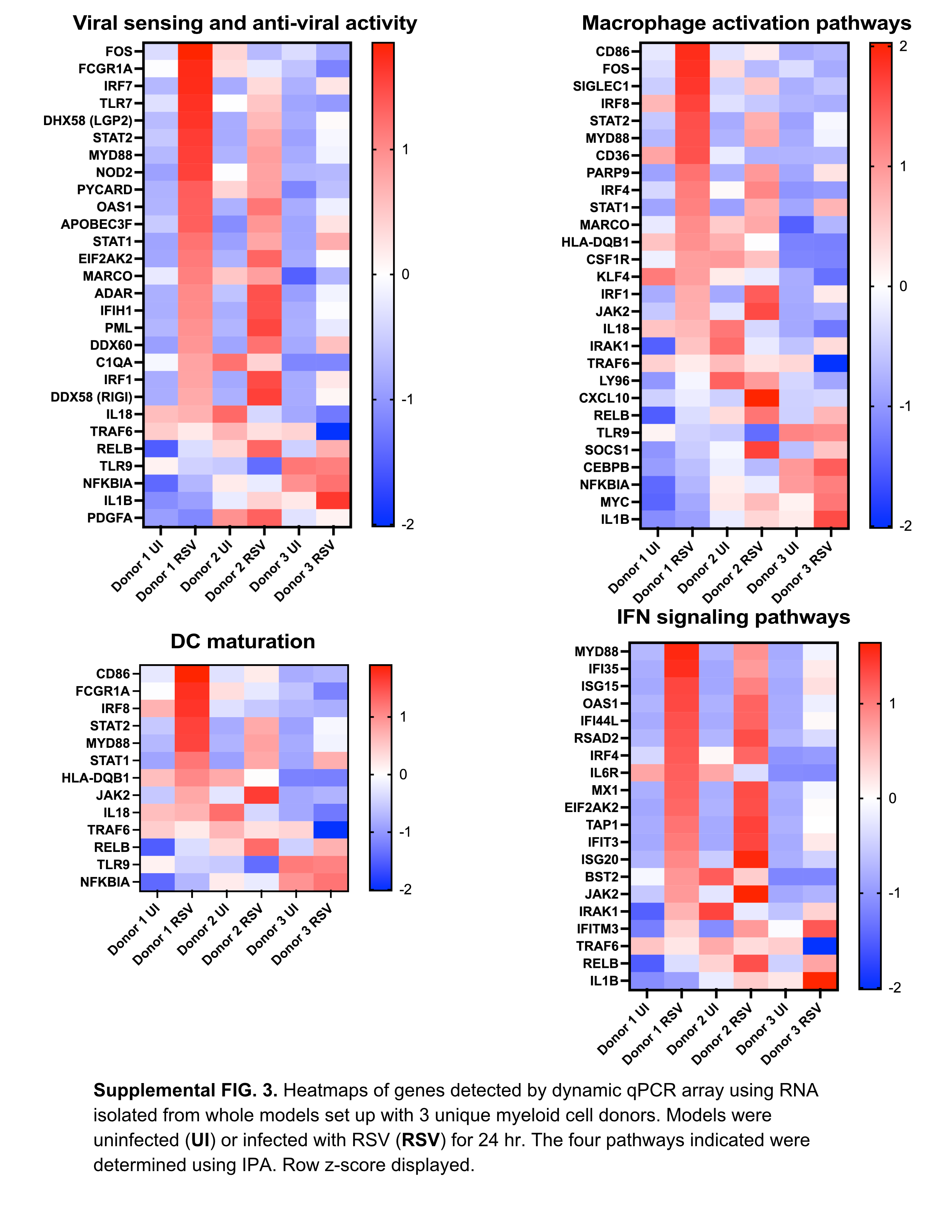

Supplement: Supplementary file 1 [file Image3.tiff]

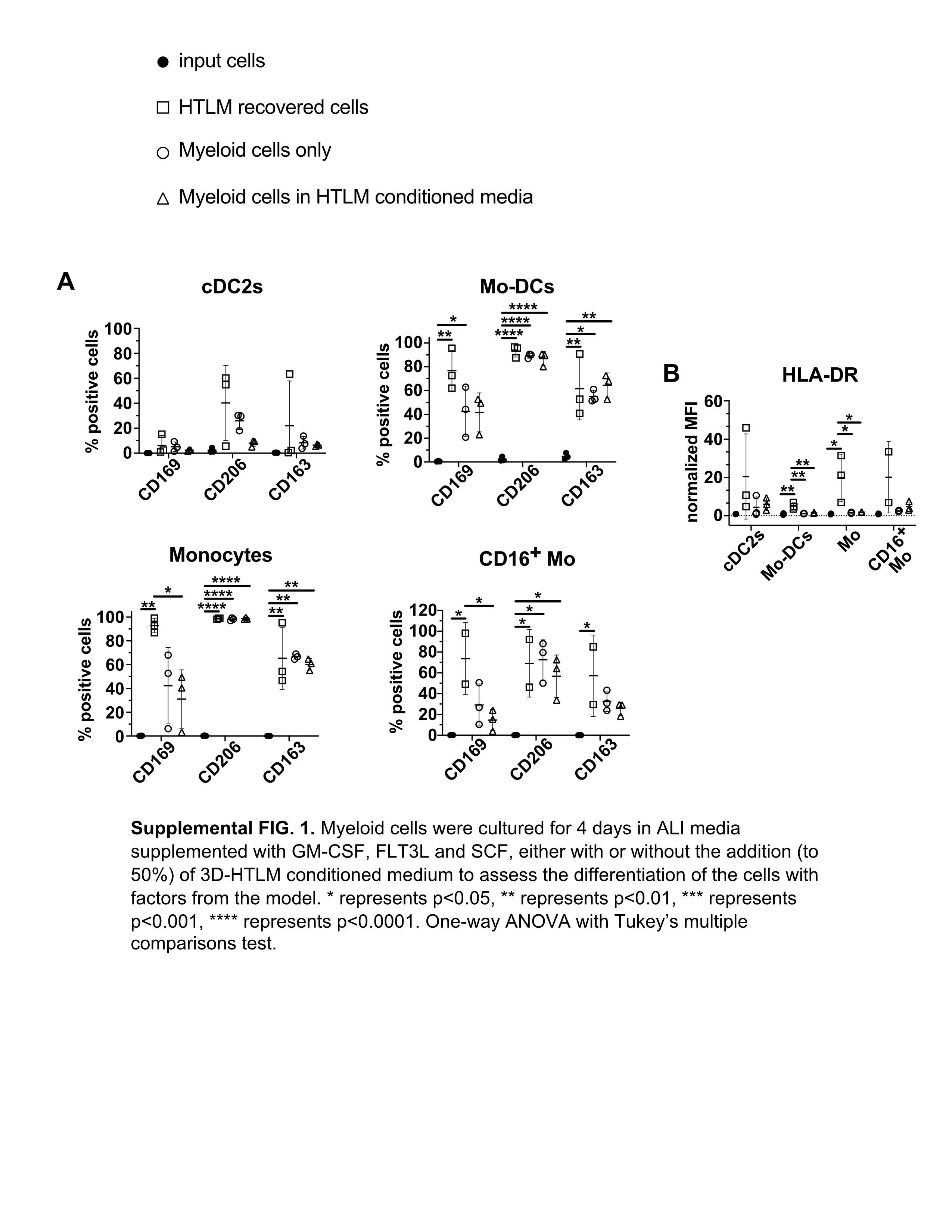

Supplement: Supplementary file 2 [file Image1.tiff]

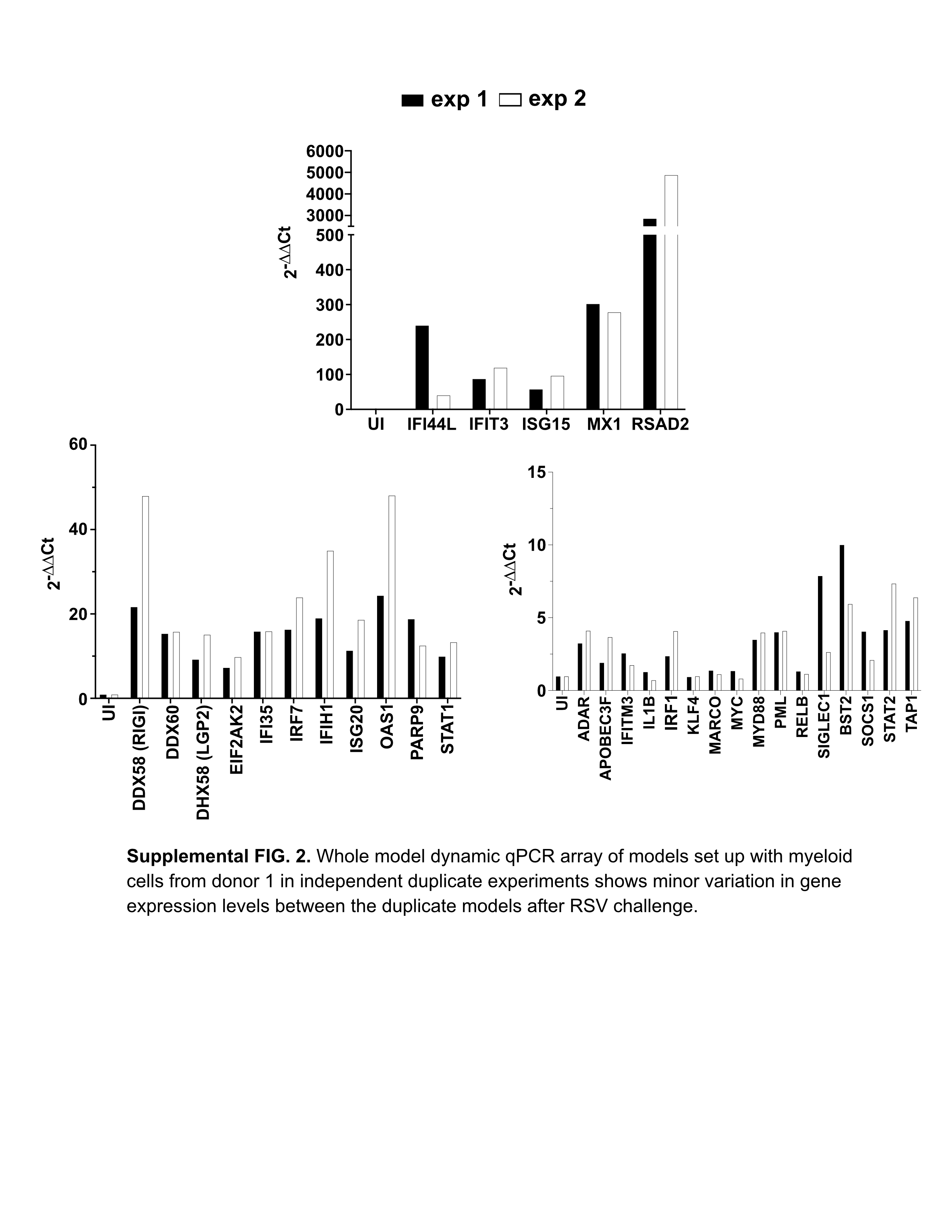

Supplement: Supplementary file 4 [file Image2.tiff]
