# Supplementary material for: Blood myeloid cells differentiate to lung resident cells and respond to pathogen stimuli in a 3D human tissue-engineered lung model
Source: Front Bioeng Biotechnol. 2023 Jul 7;11:1212230. doi: 10.3389/fbioe.2023.1212230 (PMC10361305; doi:10.3389/fbioe.2023.1212230)
Supplement: Supplementary file 3 [file Table1.docx]

| **Table S1. Antibodies used in this study** | | | | |
| --- | --- | --- | --- | --- |
| **Antibody** | **Clone** | **Fluorophore** | **Manufacturer** | **Cat #** |
| CD14 | M5E2 | BV605 | Biolegend | 301834 |
| CD14 | Polyclonal | Unconjugated | Abcam | 45870 |
| CD16 | 3G8 | PerCP/Cyanine5.5 | Biolegend | 302027 |
| CD16 | 3G8 | PE | Biolegend | 302056 |
| CD163 | GHI/61 | APC/Fire 750 | Biolegend | 333633 |
| CD169 | REA1176 | APC | Milteny Biotec | 130-121-116 |
| CD169 | HSn 7D2 | Unconjugated | Novus Biologicals | NB600-543SS |
| CD1c | L161 | PE-Cy7 | Biolegend | 331516 |
| CD206 | 15-2 | PerCP-Cy5.5 | Biolegend | 321122 |
| CD31 | WM59 | Unconjugated | Biolegend | 303102 |
| CD40 | 5C3 | BV421 | Biolegend | 334331 |
| CD40 | 5C3 | APC/Fire 750 | Biolegend | 334344 |
| CD45 | HI30 | BV785 | Biolegend | 304048 |
| CD45 | Polyclonal | Unconjugated | Novus Biologicals | NBP1-88103 |
| CD71 | CY1G4 | BV421 | Biolegend | 334121 |
| CD86 | IT2.2 | PE/Dazzle 594 | Biolegend | 305434 |
| CK-14 | LL001 | Unconjugated | Santa Cruz Biotec | 53253 |
| DkαGt | Polyclonal | AF488 | Jackson Immuno-Research Labs | 705-546-147 |
| DkαMs | Polyclonal | AF594 | Jackson Immuno-Research Labs | 715-586-151 |
| DkαRb | Polyclonal | AF488 | Jackson Immuno-Research Labs | 711-546-152 |
| FcεR1 | AER-37 (CRA-1) | BV711 | Biolegend | 334637 |
| HLA-DR | L243 | BV421 | Biolegend | 307636 |
| HLA-DR | L243 | PE/Dazzle 594 | Biolegend | 307653 |
| ICAM | HCD54 | APC | Biolegend | 322712 |
| PDL1 | 29E.2A3 | APC | Biolegend | 329707 |
| PECAM | WM59 | PerCP/Cyanine5.5 | Biolegend | 303131 |
| RbαMs | Polyclonal | PE | Santa Cruz Biotec | 516141 |
| VCAM | STA | PE | Biolegend | 305805 |

| **Table S2. Primer/Probe for RT-qPCR of RSV F protein** | |
| --- | --- |
| **Forward primer sequence** | AACAGATGTAAGCAGCTCCGTTATC |
| **Reverse primer sequence** | CGATTTTTATTGGATGCTGTACATTT |
| **Probe sequence** | TGCCATAGCATGACACAATGGCTCCT |

| **Table S3. Primers used for WISH assay** | | |
| --- | --- | --- |
| **Gene name** | **Forward primer sequence** | **Reverse primer sequence** |
| *IFIT1* | CTCCTTGGGTTCGTCTATAAATTG | AGTCAGCAGCCAGTCTCAG |
| *MX1* | TACCAGGACTACGAGATTG | TGCCAGGAAGGTCTATTAG |
| *PRKR* | CTTCCATCTGACTCAGGTTT | TGCTTCTGACGGTATGTATTA |

| **Table S4. Primers used for high throughput PCR** | | |
| --- | --- | --- |
| **Gene name** | **Forward primer sequence** | **Reverse primer sequence** |
| *ADAR* | GCACTGTTGACCCACTTCC | CAGATGCCCTTGGCTGAAAA |
| *APOBEC3F* | GCCTGGAGCAGAAAGTGAAA | GAAGTGAGGCTTCATCCTTGG |
| *BST2* | ACATTAAACCATAAGCTTCAGGAC | GCGATTCTCACGCTTAAGAC |
| *C1QA* | AGCATCCAGTTGGAGTTGACA | ACAGAGCACCAGCCATCC |
| *CD36* | AGCAGCAACATTCAAGTTAAGCA | GCGTCCTGGGTTACATTTTCC |
| *CD86* | TCAGCTTGTCTGTTTCATTCCC | GCGTCTTGTCAGTTTCCAGAA |
| *CEBPB* | TTCCTCTCCGACCTCTTCTCC | CAGGCTCACGTAGCCGTAC |
| *CSF1R* | AGGTGATGTCCATCAGCATCC | TGCAGGCACCAGTGTCAA |
| *CXCL10* | GCTGTACCTGCATCAGCATTA | CTGGATTCAGACATCTCTTCTCAC |
| *DDX58* | GACTGGACGTGGCAAAACA | CTCCACTGGCTTTGAATGCA |
| *DDX60* | CCATGTGCTGCACTAACAAC | GGATGCTTTCTCGAGGTGAA |
| *DHX58* | CACCCACCATGTCAATGTGAAC | TGTTGATGACCACAGGATCCC |
| *EIF2AK2* | GCGAACAAGGAGTAAGGGAA | AGAGGTCCACTTCCTTTCCA |
| *FCGR1A* | AGCAGCTCTACACAGTGGTT | ACTGGCAGAGGTGATTCTGT |
| *FOS* | CCCGCAGACTCCTTCTCC | TGGTCGAGATGGCAGTGAC |
| *GAPDH* | GAACGGGAAGCTTGTCATCAA | ATCGCCCCACTTGATTTTGG |
| *GBP5* | ATGAACAAGCTGGCTGGGAA | CCAAATTCCCTTGGTGTGAGAC |
| *GUSB* | CATCGATGACATCACCGTCAC | ACAGGTTACTGCCCTTGACA |
| *HLA-DQB1* | AAGAAGGCTTTGCGGATCCC | GCACATGCCCTTAAACTGGAA |
| *HPRT1* | GCTTTCCTTGGTCAGGCAGTA | ACTTCGTGGGGTCCTTTTCAC |
| *IFI35* | ACCTGCAGCAGCTGAGAA | GGTGTGTCCTCGGAATACCA |
| *IFI44L* | GCAAAAGTGAAGCAAGTTCACA | GAACCTCACTGCAATCATCCA |
| *IFIH1* | AAAAGCATCTGAGCCTGGAAA | GCGGAAGAGCTGTTCAACTA |
| *IFIT3* | ACTGGCAATTGCGATGTACC | GCTCAATGGCCTGCTTCAAA |
| *IFITM3* | AAGGGAGGGCTCACTGAGAA | TTCATGGTGTCCAGCGAAGAC |
| *IL18* | ACCAAGGAAATCGGCCTCTA | ACCTCTAGGCTGGCTATCTTTA |
| *IL1B* | GACCTGAGCACCTTCTTTCC | CGTGCACATAAGCCTCGTTA |
| *IL6R* | GTAGTGTCGGGAGCAAGTTCA | ATGTTGGCAGGCGGATCA |
| *IRAK1* | AGTGAAGCAGAGCTTCCTGAC | TGAGCACAGTAGCCAGCAAA |
| *IRF1* | AACAAGGATGCCTGTTTGTTCC | TGGGATCTGGCTCCTTTTCC |
| *IRF4* | CACCATGACAACGCCTTACC | CGAGGGGTGGCATCATGTA |
| *IRF7* | GGCAGAGCCGTACCTGTCA | ACCGTGCGGCCCTTGTA |
| *IRF8* | TGGACATTTCCGAGCCATACA | AGCAGTTGCCACGCCTA |
| *ISG15* | CTGAGAGGCAGCGAACTCA | GCTCAGGGACACCTGGAA |
| *ISG20* | ATCTACGACACGTCCACTGAC | CCACCGAGCTGTGTCCAA |
| *JAK2* | TCTGCAGTGGAGGAGATAAACC | TGCAGGAAGCTGATGCCTA |
| *KLF4* | CTGCGGCAAAACCTACACAA | CGTCCCAGTCACAGTGGTAA |
| *LY96* | CCGAGGATCTGATGACGATT | TCCCTTGAAGGAGAATGATATTGT |
| *MARCO* | GGAGCAAGGAGTAAAGGGAGAA | TCGGTTACTACTGCCGACAA |
| *MX1* | ATGCTACTGTGGCCCAGAAA | GGCGCACCTTCTCCTCATA |
| *MYC* | CCTGGTGCTCCATGAGGA | CCTGCCTCTTTTCCACAGAAA |
| *MYD88* | CTGCAGAGCAAGGAATGTGAC | TGCTGGGGAACTCTTTCTTCA |
| *NFKBIA* | CACCTCCACTCCATCCTGAA | GGTAGCCATGGATAGAGGCTAA |
| *NOD2* | AAATCAGGTTGCCGATCTTCA | GCCAATCCATTCGCTTTCAC |
| *OAS1* | TACCCTGTGTGTGTGTCCAA | AGAGGACTGAGGAAGACAACC |
| *PARP9* | TGTCCAGGGCCACATTGAA | TGCCACAGGTCCAACTGTAA |
| *PDGFA* | GCCCATTCGGAGGAAGAGAA | CTGACTCCGAGGAATCTCGTAAA |
| *PML* | CCAACAACATCTTCTGCTCCA | TTGGAACATCCTCGGCAGTA |
| *PYCARD* | AGTTTCACACCAGCCTGGAA | GGTAGGACTGGGACTCCCTTA |
| *RELB* | TGCTTTCCGAGCCCGTCTA | CGGCCCGCTTTCCTTGTTAA |
| *RSAD2* | GACGGAACAGATCAAAGCACTA | TCTCCACAATTCTCACCCTCA |
| *SIGLEC1* | AGGAGGCGTGTTTGTAAGCA | TGTGGCTGCATCAGGATCAA |
| *SOCS1* | CATCCGCGTGCACTTTCA | GCTCGAAGAGGCAGTCGAA |
| *STAT1* | ATGCTGGCACCAGAACGAA | GCTGGCACAATTGGGTTTCAA |
| *STAT2* | ATTGACCACGGGTTGGAACA | AGCTGCCTCAGGTGAAACAA |
| *TAP1* | TGTCCAGTTCCAAGATGTCTCC | GGGCGTAGGGTGAATGTCA |
| *TLR7* | TCTTCAACCAGACCTCTACATTCC | AGCCCCAAGGAGTTTGGAAA |
| *TLR9* | TGCAACTGGCTGTTCCTGAA | ACAAGGAAAGGCTGGTGACA |
| *TRAF6* | TGGCAAATGTCATCTGTGAATAC | GCTGTAGGGCAGTCTAGATCA |
